# Molecular Epidemiology of Toxigenic *Clostridioides difficile* Isolated from Bulgarian Patients and the Prevalence of Hypervirulent ST1 Clone

**DOI:** 10.3390/microorganisms14030532

**Published:** 2026-02-25

**Authors:** Rumyana Markovska, Georgi Dimitrov, Denis Niyazi, Temenuga Stoeva, Kalina Mihova, Lyudmila Boyanova

**Affiliations:** 1 Department of Medical Microbiology, Medical University of Sofia, 1431 Sofia, Bulgaria; georgi.dimitrov.swy@gmail.com (G.D.); l.boyanova@hotmail.com (L.B.); 2 Department of Microbiology and Virology, University Multiprofile Hospital for Active Treatment “Saint Marina”, Medical University of Varna, 9002 Varna, Bulgaria; denis.niyazi@gmail.com (D.N.); temenuga.stoeva@abv.bg (T.S.); 3 Molecular Medicine Centre, Medical University of Sofia, 1431 Sofia, Bulgaria; kalina_mihova@abv.bg

**Keywords:** Bulgaria, ST1, *C. difficile*, CDI

## Abstract

The aim of the study was to investigate the MLST types and genes encoding *Clostridioides difficile* toxins from fecal clinical samples of patients from two large Bulgarian cities. Overall, 100 toxigenic isolates were obtained during a 10-year period from five hospitals in Sofia and Varna, Bulgaria. Toxin gene patterns of the isolates were determined with conventional polymerase chain reaction, and multilocus sequence typing (MLST) types were determined according to Griffith’s scheme. The evolutionary relatedness of ST types was analyzed through PHYLOViZ. Binary toxin-positive isolates accounted for 35% (35/100), and the most prevalent sequence type (ST) type was ST1 (clade2) with 34%. One binary toxin-positive isolate belonged to ST11 (clade5). The *cdt-*negative isolates were of two clades: clade 4 (ST37), with 1 *tcdA*-*tcdB*+ isolate, and clade 1, with 64 isolates. All the *cdt-*positive isolates showed 18 bp deletions of *tcd*C, except a ST11 isolate exhibiting a 39 bp deletion. Importantly, there was more than a 6-fold increase in ST1 isolates in the 2020–2023 period versus 2014–2019. The main *cdt-*negative isolates were ST3 (14%), ST2 (6%), ST42 (5%), and ST92 (5%). They were *tcdA*+*tcdB*+. Four main clonal complexes (with one allele difference) were found. The first one encompassed 19 isolates and included ST3 and ST42; the second included ST8, ST16, ST52, ST92 and ST36 (11 isolates); the third consisted of 13 isolates (ST2, ST49, ST13 and ST110 clones); and the fourth included ST10 and ST44 (5 isolates). The significance of our work is the detection of a high frequency of hypervirulent isolates during the COVID-19 pandemic period, which we attribute to the national consumption of systemic antibiotics, which increased during the second period, unlike the trend in other European countries. The results highlight the need for enhanced infection control measures and strict compliance with antibiotic stewardship programs.

## 1. Introduction

*Clostridium difficile *is a Gram-positive, spore-forming, anaerobic bacterium in the form of a thick rod and with a size up to 4–5 µm. *C. difficile* is peritrichous, motile and does not form a capsule, but it has a newly described protein S layer, a common structure for some bacteria and virtually all Archaea. In *C. difficile*, the S layer is composed of different proteins that form a two-dimensional structure. This structure is supposed to be associated with many functions, such as lysozyme resistance, bacteriophage adhesion and others [1]. *C. difficile* was discovered in 1935 as part of the normal microbiota of a healthy individual and was named *Bacillus difficile*. Later, it was renamed *Clostridium difficile* [2]. Over the next several decades, researchers observed its pathogenicity, and in 2017, it was renamed again as *Clostridioides difficile*, taking into account its differences from the *Clostridium* genus [3]. Now, *C. difficile* is classified in the order *Eubacteriales*, family *Peptostreptococcaceae* and genus *Clostridioides* [3]. Since 1978, it has been considered the cause of antibiotic-associated diarrhea. Its clinical presentations vary from asymptomatic colonization to mild or severe CDI, with watery or bloody diarrhea, mucus, leukocytosis, abdominal cramps, and fever in some cases [4,5]*. *For some patients, CDI can lead to pseudomembranous colitis and complications such as ileus, megacolon or bowel perforations. The most important risk factors for development of infection are antibiotic usage (mainly clindamycin, cephalosporins, and fluoroquinolones), prior hospitalization, older age, and proton pump inhibitor usage, among others. Antibiotic exposure disrupts equilibrium of the gut microbiota and stimulates the germination of *C. difficile* spores and their growth [4,5]*.*

The major virulence factors in *C*. *difficile* strains are two homologous large protein toxins, TcdA (~300 kDa) and TcdB (~270 kDa). Only toxigenic isolates can cause CDI, whereas nontoxigenic *C*. *difficile* strains also exist, colonizing human and animal large intestines, but do not cause diarrhea. The TcdA and TcdB toxins are encoded by the *tcdA* and *tcdB* genes, respectively, which are part of a pathogenicity locus (PaLoc). PaLoc also includes regulatory genes such as *tcdR*, *tcdC*, and *tcdE* [5]. TcdA and TcdB have enterotoxic and cytotoxic activity. They are glucosyltransferases that glycosylate and inactivate the human Ras/Rho family of guanosine triphosphatases (GTPases*),* resulting in actin cytoskeletal damage, cell rounding and apoptosis, disruption of tight junctions between enterocytes, induction of IL-1, IL-8 and TNFα secretion, increased inflammation and release of fluids into the intestines [5].

For decades, CDIs have been linked to healthcare-associated infections (HAIs), transmitted from hospitalized patients and hospital surfaces. In the 21st century, there is growing evidence for CDIs among individuals without prior antibiotic usage or hospitalization and within the community, which can be explained by the appearance of hypervirulent *C. difficile* strains [4,5]. The most important difference is that these strains can cause CDIs regardless of the risk factors. Hypervirulent isolates produce a binary toxin , *C. difficile* transferase (CDT) encoded by two (*cdtA* and *cdtB*) genes, and may also have a deletion in negative repressor* tcdC, *leading to increased production of TcdA*/*TcdB. CDT functions are not fully understood, but the toxin can cause actin cytoskeleton depolymerization, thus leading to the formation of protrusions, which enhance *C. difficile* adhesion and colonization. In addition, these strains show an increase in the production of TcdA/TcdB due to deletions in the *tcdC* gene and have cytotoxic activity [5,6,7]. CDI is associated with a high rate of recurrence, especially when the infection is caused by hypervirulent strains. Between 20 and 35% of patients suffer from recurrence, usually within 30 days after the first episode [6,8]. Furthermore, worldwide dissemination of hypervirulent *C. difficile* BI/NAP1/RT027 clone has led to increased incidence of hospital and community-acquired CDI during recent years [4,9,10,11]. In addition, the increased frequency of this clone has been linked to high morbidity and mortality rates of patients. Recent evidence of the wide distribution of *C. difficile* spores in the environment (surfaces, food, meat products, and root vegetables) and in companion animals [12,13,14], highlights the problem of CDI infections.

As a result,* C. difficile *has been included in the list of microorganisms at the “urgent” threat level by the Centers for Disease Control and Prevention [15], highlighting the need for immediate and aggressive action to prevent complications and recurrences of this infection.

In addition, CDI treatment is a challenge, since only three main antibiotics (fidaxomicin, vancomycin and metronidazole) [16] and a monoclonal antibody Benzlotoxumab [17] can be used for CDI therapy. During the COVID-19 pandemic, with excessive antibiotic usage and a decline in immunity caused by SARS-CoV2, the significance of CDI increased, creating an urgent need for measures to solve the problem.

The global distribution of strains with various toxigenic profiles has been reported, with trends differing between geographical regions [10]. Genotyping of strains plays an important role in the identification of epidemiological clusters and changes in epidemiology. PCR ribotyping, which compares intergenic spacers between 16S and 23S ribosomal RNA genes, is widely used to categorize *C*. *difficile* lineages and detect epidemic *C. difficile* strains [18]. Other methods include multilocus sequence typing (MLST), based on allelic variations of housekeeping genes, and whole genome sequencing analysis [18]. In recent decades, MLST has increasingly been recognized as a useful molecular typing method with some advantages, such as simplicity of interpretation and a possible interlaboratory comparison of test results [19]. For *C. difficile*, Griffiths et al. [19] developed an MLST scheme, including a set of seven housekeeping genes, namely *adk* (adenylate kinase), *atpA* (ATP synthase subunit alpha), *dxr* (1-deoxy-D-xylulose 5-phosphate reductoisomerase), *sodA* (superoxide dismutase), *recA* (recombinase A), *glyA* (serine hydroxymethyltransferase) and *tpi* (triose phosphate isomerase). The internet-accessible database allows simple submission and recognition of allele sequences and their combinations, thus defining MLST types [19].

The aim of our study was to determine the MLST patterns and toxin-encoding genes of *C. difficile* isolates from Bulgaria.

## 2. Material and Methods

### 2.1. Study Design, Bacterial Isolates and Toxin Detection

In the present study, we included 192 patients whose fecal samples were unformed. The stool samples were obtained during a 10-year period (2014–2023) from hospitalized patients in four hospitals in Sofia and one in Varna, as well as from ambulatory patients, investigated at the microbiology laboratory of the Department of Medical Microbiology, Medical University (MU)-Sofia. Only one sample per patient was included.

The fecal samples were tested for glutamate dehydrogenase by the chemiluminescent immunoassay (CLIA) method using the Diasorin Liaison XL apparatus (DiaSorin, Via Crescentino, Saluggia, Italy) and the LIAISON *C. difficile* GDH kit (DiaSorin, Via Crescentino, Saluggia, Italy), according to the manufacturer’s instructions, or an immunochromatographic test (RIDA QUICK *Clostridium difficile* Toxin A/B, Darmstadt, Germany), according to the producer’s recommendations.

### 2.2. Cultivation and Susceptibility Testing

At the microbiology laboratory of the Department of Medical Microbiology, MU-Sofia, all fecal samples were subjected to an ethanol shock method prior to inoculation. Equal amounts of the fecal sample and 95% ethanol were mixed, homogenized, and incubated for 30–60 min at room temperature. Thereafter, the samples were plated onto a selective medium (*Clostridium difficile* agar base, Liofilchem, Roseto degli Abruzzi, Italy) with 5% horse blood and a selective supplement (Oxoid, Basingstoke, UK) containing 12 mg/L norfloxacin and 32 mg/L moxalactam, as well as onto a non-selective Anaerobe Basal Agar (Thermo Fisher Scientific, Basingstoke,, UK) with hemin, vitamin K and 5% horse blood. Colonies suspicious of *C. difficile* were identified by RapID ANA II (Remel, Inc. (Part of Thermo Fisher Scientific), Lenexa, KS, USA) or MALDI TOF MS (Vitek MS, bioMerieux, Marcy-l’Étoile, France). At the laboratory of microbiology and virology in Varna, fecal samples were cultured on ready-made Petri dishes with the selective CHROMID CDifficile agar (bio-Mérieux, France). After 48–72 h of anaerobic cultivation, the suspected colonies (colonies with a brown to black color and a specific odor) were Gram-stained and subcultured on Petri dishes with *C. difficile* CLO blood agar (bio-Mérieux, France). Identification was performed by MALDI TOF MS, Bruker, Germany. The confirmed *C. difficile* strains were sent to the laboratory of the Department of Medical Microbiology, MU-Sofia, for susceptibility testing of the isolates. Metronidazole and vancomycin susceptibility were tested by Liofilchem^®^ MIC Test Strips (Liofilchem, Italy) and that of fidaxomicin was assessed by a breakpoint susceptibility testing method, as reported previously [20]. The results were read according to EUCAST breakpoints [21].

### 2.3. Genetic Analysis

*C. difficile* DNA was extracted from the grown colonies using thermo extraction. Briefly, the bacteria were harvested from the agar in Tris-EDTA (TE) buffer and washed once with TE buffer. The pellet was resuspended in TE buffer and heated at 100 °C for 10 min. The thermo extracts were stored at −20 °C until being used for PCR analysis.

PCR was performed using primers for *gluD* (glutamate dehydrogenase as a marker of *C. difficile*) [22], *tcdA* (toxin A) and *tcdB* (toxin B) genes [23,24]. For the binary toxin, a multiplex PCR was used for detection of *cdtA/cdtB* genes according to the protocol of Persson et al. [25]. *TcdA* and *tcdC* deletions were detected with primers and protocols reported by Kato et al. [24] and Persson et al. [25].

### 2.4. MLST Typing

The primers and protocols were previously described [19]. They included amplification of the 7 housekeeping genes (*adk*, *atpA*, *dxr*, *glyA*, *recA*, *sodA*, *tpi*) for each isolate. Then, the amplicons were sequenced with Sanger sequencing. The assignment to allelic numbers and sequence types (STs) was performed according to the PubMLST database [https://pubmlst.org/organisms/clostridioides-difficile accessed on 20 December 2025]. The evolutionary relatedness among ST types of *C. difficile* isolates was analyzed through the online website PHYLOViZ, [https://online.phyloviz.net/index, accessed on 2 January 2026].

### 2.5. Statistical Analysis

Differences between the groups were assessed by the chi-square test or Fisher’s exact test using GraphPad [https://www.graphpad.com/quickcalcs/contingency1/ accessed on 2 January 2026]. The differences were considered significant when the *p* value was <0.05.

## 3. Results

### 3.1. Characteristics of Patients and C. difficile Toxigenic Isolates and Their Susceptibility

Fecal samples positive for TcdA/B and GDH were cultivated. On selective *C difficile* blood agars, the isolates yielded colonies of varying sizes with diameters between <1 and 4–5 mm. They were non-hemolytic, grey/whitish, flattish, and irregular-edged, and on CDChromid, the colonies had brown to black colors and a specific horse manure odor. Gram staining revealed that the microorganisms were Gram-positive rods with or without subterminal spores (after prolonged cultivation). The MALDI-TOF MS or RapID ANA II confirmed that microorganisms were *C difficile*. The isolates were further examined for *gdh* and *tcdA/tcdB* with PCR. As result, a total of 100 C. *difficile* isolates positive for *tcdA/tcdB* from 100 nonduplicated patients were included in the study.

The patients had symptoms of diarrhea and were clinically diagnosed with enterocolitis/possible CDI, and two patients had pseudomembranous colitis. Depending on the patient’s age, the isolates were divided into four groups: ≤2 years (14 isolates), 2 to 16 years (15), 17 to 64 years (35), and ≥65 years (36). The mean age was 45.5 years, ranging from 2 months to 91 years. Males comprised 48% and females comprised 52% of the patients. Ninety-two patients were hospitalized in four hospitals in Sofia and one hospital in Varna, and eight were ambulatory (in Sofia). In Table 1 we present the patients’ characteristics and comorbidities. All the isolates were susceptible to fidaxomicin, vancomycin and metronidazole, except seven isolates resistant to metronidazole (7%), three to vancomycin (3%) and one to fidaxomicin.

### 3.2. Genetic Analysis: Toxigenic Characteristics and tcdA and tcdC Deletions

All isolates were positive for *gdh*, thus proving their species identification. The majority of isolates had the toxigenic genotype *tcdA*+ *tcdB*+ *cdtA/B*− (65/100, 65%), followed by *tcdA*+ *tcdB*+ *cdtA/B*+ (35 isolates). One isolate tested positive for *tcdA*, but with primers NK9 and NK11 [24], it showed an amplicon of 600 bp, indicating a deletion in *tcdA* at its 3′ repetitive terminus. All the other isolates had amplicons of 1200 bp, showing intact *tcdA* (no gene deletion*)*. Thirty-four isolates had an 18 bp deletion in *tcdC* (producing an amplicon with a 144 bp size) and one had a 39 bp deletion (producing an amplicon with a 126 bp size) . 

### 3.3. MLST Typing

All 100 strains were classified into 25 STs (Table 2), comprising four main clades (clades 1, 2, 4, and 5). The most prevalent ST was ST1, clade 2 (*n* = 34). All isolates of this ST type had the genotype pattern *tcdA*+ *tcdB*+ *cdtA/B*+. ST3 (*n* = 14), ST42 (*n* = 5), and ST92 (*n* = 5) were from clade 1, and they were *cdt-*negative. All ST1 and ST11 isolates showed deletions in *tcdC*, with 18 bp for ST1 and 39 bp for ST11. The isolates of the other clones had no deletions in *tcdC*. The ST37 isolate exhibited a truncated *tcdA* gene with a 600 bp deletion. Table 2 shows the characteristics of the different ST types.

Interestingly, ST1 isolates were observed predominantly in the second period, 2020–2023, (44%, 32/72) versus 2014–2019 (7.1%, 2/28 isolates, *p* = 0.0002), and mainly among adult patients (46.5%, 33/71) vs. children (3.5%, 1/29, *p* < 0.0001). They were detected in all hospitals in Sofia and Varna and among ambulatory patients (Table 2).

Similarly, the ST3 clone was found mainly in the second period 2020–2023, with 11/72 (15.2%) versus 3/28 (10.7%) in the first period (Table 2), and among adults (18.3%, 13/71) versus children (3.4%, 1/29) (Table 2); however, the differences were not statistically significant.

**Figure 1:**
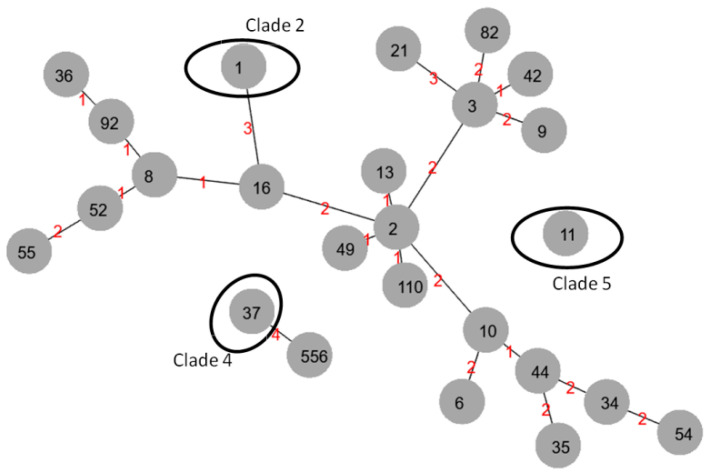
Minimal spanning tree showing the evolutionary relatedness of the different *C. difficile* ST types. Legend: The grey circles show the ST numbers. The big black circles mark isolates from the spe-cific clades (2,4,5). All other ST types belonged to clade 1. The red numbers show the distance be-tween the ST types (numbers of different alleles).

The evolutionary relatedness of the different STs was analyzed with PHYLOVIZ (Figure 1). There were four main clonal complexes (with one allele difference): the first represented 19 isolates, including ST3 and ST42; the second encompassed a total of 11 isolates, including ST8, ST16, ST52, ST92 and ST36; the third encompassed 13 isolates of ST2, ST49, ST13 and ST110 clones; and the fourth included 5 isolates, including ST10 and ST44. All clonal complexes belonged to clade 1.

The seven metronidazole-resistant isolates belonged to ST1 (three isolates), ST2, ST6, ST44 and ST92 (one for each), three vancomycin-resistant isolates belonged to ST2, ST3 and ST6, and one isolate resistant to fidaxomicin belonged to ST3.

## 4. Discussion and Analysis

*C. difficile *is a challenge for treatment and diagnosis due to its worldwide transmission and high virulence, causing infections with significant morbidity, mortality and recurrence frequency. With more than 18.3 million CDI patient admissions in Europe in 2016–2017 [26], this microorganism represents a huge threat to the healthcare system. The current study included 100 isolates distributed in almost equal proportion between the females and males, with slightly more (52%) female patients. This is in concordance with the prevalence of females (56.4%) in an ECDC surveillance report for 2016–2017 [26]. Similar prevalence was reported by Persson et al. for Denmark [27]. Around one-third (29%) of the isolates in the current study were from children, and the other two-thirds were from adults. Interestingly, among the 71 adult patients, 35 were aged 17–64 years, and the other 36 patients were aged ≥65 years, which is in contrast with the ECDC report (for Europe in 2016–2017, reporting 72% of the CDI cases in patients aged >64 years).

Five main phylogenetic clades (clades 1–5) and four cryptic (C-I to C-IV) clades have been described. The clades correlated well with MLST types and PCR ribotypes [6,11,13]. Hypervirulent strains were also frequent in certain ribotypes and had a highly corresponding association with specific clades [28].

The present study revealed 25 MLST types among the 100 isolates, collected over a 10-year period in two towns in Bulgaria, and their association with patient characteristics, toxin genotype and resistotype profiles. Briefly, we detected 35% *cdtA/B*-positive isolates, mainly belonging to clade 2 (ST1, 34%) and clade 5 (ST11, one isolate). The other 22 ST types were binary toxin-negative and *tcdA /tcdB*-positive, all belonging to clade 1. Within clade 4, one isolate belonged to ST37 and had a truncated *tcdA*.

The most important finding was the detection of *cdt*A/B-positive isolates in 35%, with 34% belonged to the **ST1 clone**. ST1 is part of clade 2. This lineage includes the most commonly reported and hypervirulent RT027 ribotype, but also the RT176, RT181 and RT244 variants [13,28].

The *C. difficile* ST1 (BI/NAP1/027) clone first appeared in Europe and North America early in the 21st century and was named “hypervirulent” due to its ability to cause infections without the need for risk factors such as antibiotic exposure, previous hospitalization or disruption of gut microbiota.

TcdC protein is a negative regulator of *tcdA* and *tcdB* toxin production, and its inactivation allows for increased (more than 20 times) TcdA/TcdB toxin production. Increased toxin production, among ST1 isolates, has been associated with an 18 bp deletion in *tcdC* and a deletion at position 117, which has been linked to a frameshift mutation and *tcdC* truncation [4]. Overall, ST1 (027 ribotype) has been associated with increased virulence, a high frequency of complications, more severe cases of CDIs, high mortality, and high recurrence rates [4,5,29].

The emergence of the hypervirulent RT027 *C. difficile* strain has substantially contributed to the rise in CDI incidence not only in hospitals, but also within the community [11]. A large increase in RT027 incidence and more complications of CDI have been observed since March 2003 in Canada, the USA and some European countries (including England, Netherlands, Belgium, and France) [30]. This RT type has steadily been spreading in Europe and America, whereas in Asia and Africa, it has been detected in only single cases [31], apart from its high prevalence in South Africa [32]. One of the important waves happened during the period 2012–2013, predominantly in Germany and Eastern Europe (Hungary, Poland and Romania), in which 22% of isolates belonged to RT027 according to a study by Davies et al. [33]. Eyre et al. [34] reported, for the same period, prevalence of RT027 in Hungary, Italy, Germany, Romania and Poland. Freeman et al. [35] also found wide dissemination of RT027 in Europe in 2011–2016. In a Polish study, one of the highest percentages of RT027 isolates (77.2%) was found [36]. The ST1 lineage shows great diversification, and in addition to RT027, it also includes RT176 and RT181. RT176 was reported in Slovakia [37], Czech Republic [38] and Poland [36]. RT181 was detected in Greece [39]. The RT027-like ribotypes were hypervirulent and characterized by *cdtA/B* positivity and 18 bp deletion in *tcdC* [38,39]. In Europe, with the introduction of antibiotic stewardship and strong infection control measures, CDI frequency has decreased, together with a decline in RT027 frequency [40]. Pan-European studies for 2018 [12] and the ECDC report for 2016–2017 [26] showed a change in the main ST types, and the frequency of RT027 (ST1) moved to the second and third places, respectively. In Germany, Abdrabou et al. [41] reported a significant decrease in RT027 from 36.2% in 2016 [42] to about 3.5% in 2019–2021. Similarly, Persson et al. [27] reported a temporal decline of hypervirulent ST1 clone in Denmark during the 2016–2019 period.

Interestingly, in the current study, we observed only two (7.1%) ST1 isolates in the period 2014–2019, in contrast to the period 2020–2023, when 44% were detected. This is more than a six-fold increase, and the difference is statistically significant. Thus, our results are in contrast to those from the previously mentioned European studies, as well as studies such as the COMBACTE-CDI point-prevalence study for 2018 [11], and the ECDC surveillance report for 2016-2017, in which a decrease in RT027 frequency was observed since 2016 [26]. However, the overall prevalence of RT027 in the current study (34%) was lower than those found in some Eastern European countries such as Hungary (67.6%), Poland (63.0%) and Slovenia (44.4%) in 2016/2017 [26]. The increase in ST1 during the second period in our study may have been associated with the fact that 2020–2023 was the period of the COVID-19 pandemic. In contrast to many European countries, in which antibiotic usage decreased between 2019 and 2022, Bulgaria had the highest increase, of 27%, in the total consumption (in the community and hospital sectors) of systemic antibacterials [43]. Antibiotic consumption was 26.3 DDD per 1000 inhabitants per day in Bulgaria in 2023, and our country ranked second regarding the consumption of systemic antibacterials [43], following Greece. In addition, in Bulgaria, one of the highest frequencies (61%) of cephalosporin and carbapenem usage was observed in 2019–2022 [44].

Interestingly, two observational studies have previously been published for Bulgaria. The first one was for the period 2008–2012 [45], and the second was for 2015–2022 [46]. In the first one, the authors did not find the presence of RT027, while in the second period, 33.3% of the isolates (mainly from the 2020–2022 period) were ST1 (RT027), similar to the data in our study (34%). Dobreva et al. [46] found that Bulgarian ST1 (RT027) isolates clustered together with isolates from Hungary, Spain, Romania and Australia. In the current study, six ST1 isolates were from COVID-19 patients (in total, 12 isolates were from COVID-19 patients). The reason for this could be higher intrahospital transmission of *C. difficile* during the COVID-19 pandemia, which is characteristic for RT027 [11]. In fact, Dobreva et al. [46] reported that almost all COVID-19 patients had ST1 or ST3 isolates [46]. Some Bulgarian authors reported that the COVID-19 pandemic increased the frequency of CDI by 21.9% from 2020 to 2022. [47]. The authors found an increase of 22% during the COVID-19 period [47].

Interestingly, three of the seven metronidazole-resistant isolates (MICs, >2mg/L) belonged to the ST1 lineage. No vancomycin- or fidaxomicin-resistant isolates were detected in this group. Many authors, including those from Poland [36], Serbia [48], and the USA [49], have reported a lack of resistance to metronidazole, fidaxomicin and vancomycin among ST1 isolates, although this clone has been associated with resistance to mainly quinolones and rifampicin and sometimes clindamycin, macrolides and cephalosporins [36,48,50,51]. Some of these antibiotics can act as drivers of *C. difficile* intrahospital dissemination [34,51,52]. However, metronidazole resistance did exist and was associated mainly with ST1 and ST11 variants. In Germany, metronidazole resistance frequencies of around 5.9% in 2014-19 and 3.3% in 2019–21 were observed [41,42], while the mean resistance rate in one review was 2.6% [51].

In the current study, we observed only one isolate of the **ST11 clone**, for which RT078 is the most common ribotype. This isolate belonged to the hypervirulent *cdt*A/B-positive lineage, with a 39 bp deletion in *tcd*C [4,13,19]. ST11 belongs to clade 5, its major reservoirs are animals and livestock, and through animal-to-human transmission, it can cause human infections. It has a low probability of hospital transmission and causes community-acquired infections [34]. This lineage showed high antimicrobial resistance levels, due to many antimicrobial resistance determinants, which may contribute to its persistence in the environment and its global transmission [11,12,53]. ST11 is heterogenous and includes many RT types, such as RT078 and RT126 (most often), as well as RT033, RT045, and RT066. RT078 was widely disseminated in Europe, and was the second most common isolated *C. difficile* RT in human CDI in Europe in 2016–2017 [26]. It is commonly derived from production animals, farm animals (pigs) and farmworkers, as well as from soil and water [11,27,31,54]. Our isolate was *tcdA/B* positive and *cdtA/B*-positive with a 39 bp deletion in *tcdC* and not resistant to vancomycin, metronidazole or fidaxomicin, similar to other results [12]. However, the ST11 clone is specifically associated with tetracycline resistance [12,51]. This clone was observed in the first period of the current study and aligns with the results of Dobreva et al. published in 2013 and 2025 [45,46]. Similarly to our study, the authors detected only one RT078 (ST11) isolate in the 2012–2013 period [45], but not in 2015–2022.

Another interesting isolate in our study was **ST37** from clade 4, with the main ribotype RT017. This lineage can be found on every continent, but is endemic in Asian Pacific region and predominant in China [31,50,55]. ST37 (RT017) is one of a few types producing only TcdB and has been associated with several outbreaks in the world, mainly in Asian countries, but also in Australia, the USA, Canada and Germany [31,50]. Despite the presence of truncated nonfunctional *tcdA*, most of the *tcdA* gene in *C. difficile* RT017 (ST37) remain intact and can be detected by PCR [24]. This is why, in order to avoid false positive results, we used a second primer set that can detect deletion in *tcdA*. Despite having a truncated *tcdA*, *C. difficile* RT017 strains caused disease as severe as those by strains producing both A and B toxins and binary toxin. In addition, many reports have also mentioned higher mortality of ST37 isolates and its resistance to several antibiotics [50]. In an interesting study, the ST37 (RT017) strain was found to affect macrophage vitality [56]. In concordance with these studies, the single isolate of the ST37 clone in the current study was isolated from a COVID-19 patient.

In clade 1, the prevailing clone in our collection was **ST3**. ST3 is a highly heterogenous cluster, with RT001 being its most common ribotype. ST3 was found in 14% of isolates in this study. Most of them were from the second (COVID-19) period and from adult patients; however, the differences were not statistically significant. The next clone was **ST2**, corresponding to RT014/020, detected in 6% of our isolates. ST3 (RT001) and ST2 (RT014/020) are a worldwide distributed lineages. They were observed in Europe (RT001 prevailed in 2011 and RT014 predominated in 2014) [31,35,41,42]. Eyre et al. [34] found RT001 to be the dominant RT in Germany and Slovakia for 2012/2013. In the period 2016–2019, Persson observed that RT014/020 (ST2/13) was dominant at 19.5% [27]. In 2016/2017, ECDC reported that RT014/020 was the most distributed clone, comprising 16.8% of cases [26]. In general, in Europe and North America, after decreases in the RT027 and RT027-like lineages, the prevailing clone was ST2 (RT014/020) [31]. A study from China also identified ST2 and ST3 as the most common clusters [57]. Our proportion (14% for ST3) was lower than that in another Bulgarian study, which showed ST3 (RT001) to be the predominant clone (46.1%) in 2015–2022 [46]. The reason could be linked to the different hospitals included in the studies. Dobreva et al. [46] included hospitals from Sofia, while in our study, we included hospitals from Sofia and Varna. Three of the ST3 clone isolates were from COVID-19 patients. Dobreva et al. [46] also associated COVID-19 patients mainly with ST1 and ST3 clones [46].

ST2 (RT014/020), common in Europe and America [31], was found in only six isolates in our study. Interestingly, from the six ST2 isolates, two were resistant—one of them to metronidazole and one to vancomycin. The ST3 isolates also contained two resistant isolates (one to vancomycin and, interestingly, one to fidaxomicin). The resistance to these main antibiotics for CDI therapy is quite unusual [51,57] but does exist [4,26,31,52]. There are reports in which fidaxomicin use has led to increased frequency of resistant isolates [58]. It is important to stress that fidaxomicin is not yet available in Bulgaria; therefore, the resistant isolate appeared without selective pressure. However, due to frequent travel to countries where fidaxomicin is in use, the origin of resistance cannot be determined. Unfortunately, we do not know the travel history of this patient. Our results once again emphasize the need for cultivation and antibiotic susceptibility testing of toxigenic *C. difficile*.

The ST2 and ST3 clones showed potential for diversification since they were ancestral founders of clonal complexes: ST2 included **ST49, ST13 and ST110** (a total of 13 isolates), and ST3 had one allele difference with **ST42** (5 isolates). Interestingly, ST42 can affect macrophage vitality and increase cytokine secretion levels, including IL-12, IL-6 and TNF-α [56]. One of the reasons for the predominance of ST2 (RT014/020) could be its wide dissemination in the environment [12,58].

The other ST types included one to two isolates. The clonal complex containing **ST8** as an ancestral founder also encompassed **ST92, ST16 and ST52** (*n* = 11).

Analysis of our results showed a rapid change in the MLST types of *C difficile.* Unlike results from other countries, ST1 increased in Bulgaria during 2020–2023 (the COVID-19 pandemic period). We attribute this change to the national consumption systemic antibiotics, which increased during the second period, unlike the trend in other European countries. This is why we suggest that antibiotic policy should be implemented and strictly complied with in all countries. Another finding that differed from other studies [36,48,49] was the detection of rare fidaxomicin resistance, even though the agent is not yet available in our country. Based on our data, we suggest that the antibiotic susceptibility of the isolates is tested even before they are introduced in a given country.

## 5. Conclusions

We detected an alarmingly high frequency of hypervirulent isolates: 34% ST1 (clade 2) and 1% ST11 (clade 5). From clade 4, *cdt-*negative, one ST37 isolate was detected. All the other 22 ST types, clade 1, formed four main clonal complexes, of which ST3 and ST2 were the most common. The detection of three vancomycin-resistant and one fidaxomicin-resistant isolates highlights the need for cultivation, antimicrobial susceptibility testing, and genetic investigation of the isolates from all patients suspicious for CDI. The established high prevalence of hypervirulent isolates in the present study impose a need for strengthening infection control measures and antibiotic stewardship programs.

## Figures and Tables

**Table 1:** Patient characteristics.

Age, *n* (%)	≤16 years *	29 (29%)
	17–64 years	35 (35%)
	≥65 years	36 (36%)
Gender, *n* (%)	Males	48 (48%)
	Females	52 (52%)
Comorbidity **, *n*	Ulcerative colitis	9
	Hematologic diseases	7
	Cardio-cerebrovascular diseases	4
	Solid tumors	4
	Dementia, depression	3
	Post-COVID-19 enterocolitis	12
	Gastrointestinal non-CDI diseases	3
	Chronic kidney insufficiency, nephritis, kidney transplantation	7
	Other—pyoderma, cystic fibrosis, chronic obstructive lung disease	3

Abbreviations: * Including 14 patients between 1 and 2 years, all of them had diarrhea, which cannot be explained by reasons other than CDI. ** The underlying disease was known for 4 children, including gastritis, cystic fibrosis, allergic colitis, and ileus.

**Table 2:** MLST patterns and their association with age, period, place of isolation, toxic genotypes, and *tcdC* deletions.

ST Types, Clade ^1^	*n*	Children *n*	Adults *n*	2014–2019 *n*	2020–2023 *n*	Place of Isolation (*n*) ^2^	Toxin Genotype	*tcdC* Deletion
**ST1,cl 2**	34	1	33	2	32	Sofia (11),Varna (20), amb (3)	*tcdA* + *tcdB* + *cdtA/B*+	18-bp
ST2,cl 1	6	3	3	3	3	Sofia (4),Varna (2)	*tcdA* + *tcdB* + *cdtA/B*−	No
**ST3,cl 1**	14	1	13	3	11	Sofia (11),Varna (2), amb (1)	*tcdA* + *tcdB* + *cdtA/B*−	No
ST6,cl 1	3	1	2	1	2	Sofia (3)	*tcdA* + *tcdB* + *cdtA/B*−	No
ST8,cl 1	2	2		1	1	Sofia (2)	*tcdA* + *tcdB* + *cdtA/B*−	No
ST9,cl 1	1	1			1	Varna (1)	*tcdA* + *tcdB* + *cdtA/B*−	No
**ST10,cl 1**	3	2	1		3	Sofia (3)	*tcdA* + *tcdB* + *cdtA/B*−	No
ST11,cl 5	1		1	1		Sofia (1)	*tcdA* + *tcdB* + *cdtA/B*+	39-bp
ST13,cl 1	1		1		1	Varna (1)	*tcdA* + *tcdB* + *cdtA/B*−	No
**ST16,cl 1**	2		2	1	1	Sofia (2)	*tcdA* + *tcdB* + *cdtA/B*−	No
ST21,cl 1	1		1		1	Sofia (1)	*tcdA* + *tcdB* + *cdtA/B*−	No
ST34,cl 1	1		1		1	Varna (1)	*tcdA* + *tcdB* + *cdtA/B*−	No
ST35,cl 1	3	3		2	1	Sofia (2),Varna (1)	*tcdA* + *tcdB* + *cdtA/B*−	No
ST36,cl 1	1	1		1		Sofia(1)	*tcdA* + *tcdB* + *cdtA/B*−	No
**ST37,cl 4**	1		1		1	amb (1)	*tcdA* − *tcdB* + *cdtA/B*− ^3^	No
ST42,cl 1	5	2	3	1	4	Sofia (2),Varna (3)	*tcdA* + *tcdB* + *cdtA/B*−	No
ST44,cl 1	2	1	1	2		Sofia (2)	*tcdA* + *tcdB* + *cdtA/B*−	No
ST49,cl 1	4	2	2	2	2	Sofia (3), amb (1)	*tcdA* + *tcdB* + *cdtA/B*−	No
ST52,cl 1	1		1		1	Varna (1)	*tcdA* + *tcdB* + *cdtA/B*−	No
ST54,cl 1	3	2	1		3	Sofia (1),Varna (2)	*tcdA* + *tcdB* + *cdtA/B*−	No
ST55,cl 1	1	1		1		Sofia (1)	*tcdA* + *tcdB* + *cdtA/B*−	No
ST82,cl1	1		1	1		Sofia (1)	*tcdA* + *tcdB* + *cdtA/B*−	No
ST92,cl 1	5	3	2	4	1	Sofia (3), amb (2)	*tcdA* + *tcdB* + *cdtA/B*−	No
ST110,cl 1	2	2		1	1	Sofia (1),Varna (1)	*tcdA* + *tcdB* + *cdtA/B*−	No
ST556,cl 1	2	1	1	1	1	Sofia (1),Varna (1)	*tcdA* + *tcdB* + *cdtA/B*−	No
Total	100	29	71	28	72	Sofia (**56**), Varna (**36**), amb (8)		

Abbreviations: ^1^ ST types of isolates from COVID patients are marked in bold (6 ST1, 3 ST3 and 1 each from ST10, ST16 and ST37; ^2^ Sofia—patients from four hospitals in Sofia; Varna—patients from one hospital in Varna; ^3^ isolate ST37 had a truncated *tcdA*; *n*—number, cl—clade amb—ambulatory patients.

## Data Availability

The original contributions presented in this study are included in the article. Further inquiries can be directed to the corresponding author.
